# Evaluation of Rituximab for Induction and Maintenance Therapy in Patients 75 Years and Older With Antineutrophil Cytoplasmic Antibody–Associated Vasculitis

**DOI:** 10.1001/jamanetworkopen.2022.20925

**Published:** 2022-07-08

**Authors:** Sara Thietart, Alexandre Karras, Jean-François Augusto, Carole Philipponnet, Pierre-Louis Carron, Xavier Delbrel, Rafik Mesbah, Gilles Blaison, Pierre Duffau, Khalil El Karoui, Perrine Smets, Jonathan London, Luc Mouthon, Loïc Guillevin, Benjamin Terrier, Xavier Puéchal

**Affiliations:** 1 Department of Internal Medicine, National Referral Center for Rare Systemic Autoimmune Diseases, Hôpital Cochin, Assistance Publique–Hôpitaux de Paris, Paris, France; 2 Department of Geriatrics, Sorbonne Université, Assistance Publique–Hôpitaux de Paris, Groupe Hospitalier Pitié-Salpêtrière-Charles Foix, Paris, France; 3 Department of Nephrology, Hôpital Européen Georges Pompidou, Assistance Publique–Hôpitaux de Paris, Université de Paris Cité, Paris, France; 4 Department of Nephrology, Dialysis, and Transplantation, Centre Hospitalier Universitaire, Angers, France; 5 Department of Nephrology, Centre Hospitalier Universitaire, Clermont-Ferrand, France; 6 Department of Nephrology, Dialysis, and Transplantation, Centre Hospitalier Universitaire Grenoble-Alpes, Grenoble, France; 7 Department of Internal Medicine, Centre Hospitalier de Pau, Pau, France; 8 Department of Nephrology, Centre Hospitalier, Boulogne-sur-Mer, France; 9 Department of Internal Medicine, Louis Pasteur Colmar Hospital, Colmar, France; 10 Department of Internal Medicine, Centre Hospitalier Universitaire Bordeaux, Bordeaux, France; 11 Department of Nephrology and Renal Transplantation, Hôpital Henri-Mondor, Assistance Publique–Hôpitaux de Paris, Paris, France; 12 Department of Internal Medicine, Centre Hospitalier Universitaire Gabriel Montpied, Clermont-Ferrand, France; 13 Department of Internal Medicine, Groupe Hospitalier Diaconesses Croix Saint Simon, Paris, France; 14 Assistance Publique–Hôpitaux de Paris Centre, Université Paris Cité, Paris, France

## Abstract

**Importance:**

Older patients are underrepresented in studies of rituximab for the treatment of antineutrophil cytoplasmic antibody (ANCA)–associated vasculitis. Little is known about outcomes and adverse events associated with the use of rituximab therapy among patients 75 years and older with ANCA-associated vasculitis.

**Objective:**

To examine outcomes and adverse events associated with the use of rituximab therapy in patients 75 years and older with ANCA-associated vasculitis, specifically granulomatosis with polyangiitis (GPA) or microscopic polyangiitis (MPA).

**Design, Setting, and Participants:**

This multicenter cohort study involved 93 patients 75 years and older with ANCA-associated vasculitis from 36 university and nonuniversity hospitals in France. Data were obtained from the French Vasculitis Study Group database between January 1, 2000, and July 1, 2018, and a call for observation sent to French Vasculitis Study Group members on June 6, 2019. Data analysis was performed from November 15 to December 31, 2021. Inclusion criteria included a diagnosis of GPA or MPA according to European Medicines Agency classification criteria and receipt of treatment with rituximab after age 75 years. Patients were excluded if they were missing relevant clinical or biological data. Data on race and ethnicity were not reported because inclusion of this information was not authorized by the ethics committee.

**Exposure:**

At least 1 infusion of rituximab as induction or maintenance therapy.

**Main Outcomes and Measures:**

Occurrence of remission, relapse, drug discontinuation, death, and serious infections (including types of serious infections).

**Results:**

Of 238 patients screened, 93 were included (median [IQR] age, 79.4 [76.7-83.1] years; 51 women [54.8%]); 52 patients (55.9%) had a diagnosis of GPA, and 41 (44.1%) had a diagnosis of MPA. Thirty patients (32.3%) received rituximab as induction therapy in combination with high-dose glucocorticoid regimens, 27 (29.0%) received rituximab as maintenance therapy, and 36 (38.7%) received rituximab as both induction and maintenance therapy. The median (IQR) follow-up was 2.3 (1.1-4.0) years. Among 66 patients who received rituximab as induction therapy, 57 (86.4%) achieved remission, and 2 (3.0%) experienced relapses. The incidence of serious infection was significantly higher when rituximab was used as induction therapy vs maintenance therapy (46.6 [95% CI, 24.8-79.7] per 100 patient-years vs 8.4 [95% CI, 3.8-15.9] per 100 patient-years; *P* = .004). Most infections (12 of 22 [54.5%]) were gram-negative bacterial infections. The incidence of death was 19.7 (95% CI, 7.2-42.9) per 100 patient-years among those who received rituximab as induction therapy and 5.3 (95% CI, 1.9-11.6) per 100 patient-years among those who received rituximab as maintenance therapy.

**Conclusions and Relevance:**

In this cohort study, rituximab therapy was associated with achievement and maintenance of remission in most patients 75 years and older with ANCA-associated vasculitis. The incidence of serious infections and death was high when rituximab was used as induction therapy in combination with high-dose glucocorticoid regimens but not when rituximab was used as maintenance therapy. Efforts might focus on reducing serious infections during the first months of therapy.

## Key Points

**Question:**

What outcomes and adverse events are associated with rituximab use among patients 75 years and older with antineutrophil cytoplasmic antibody (ANCA)–associated vasculitis?

**Findings:**

In this cohort study of 93 patients 75 years and older with ANCA-associated vasculitis, induction therapy combining rituximab and high-dose glucocorticoid regimens was associated with achievement of remission but accompanied by a high rate of serious infections (46.6 per 100 patient-years). When rituximab was used as maintenance therapy, relapse rates (1.8 per 100 patient-years) and serious infection rates (8.4 per 100 patient-years) were low.

**Meaning:**

These results suggest that rituximab therapy may be associated with disease remission and prevention of relapse in patients 75 years and older but that efforts focused on reducing infections during induction therapy are needed.

## Teaser

This cohort study assesses outcomes associated with the use of rituximab as induction and/or maintenance therapy in patients 75 years and older with antineutrophil cytoplasmic antibody–associated vasculitis.

## Introduction

Antineutrophil cytoplasmic antibody (ANCA)–associated vasculitis is a systemic necrotizing vasculitis that affects predominantly small vessels, with few or no immune deposits.^1^ The disease is primarily found in the older population; its peak incidence rate occurs at ages 65 to 75 years and remains high after age 75 years.^2,3,4^ Clinical presentation differs between older and younger patients. Microscopic polyangiitis is more frequently diagnosed in the older population,^5,6,7,8^ with more frequent comorbidities, kidney involvement, and antimyeloperoxidase antibody positivity and less ear, nose, and throat involvement. Outcomes also differ, with older individuals having higher infection and mortality rates^6,9,10^ but a lower risk of relapse.^7^

For the treatment of ANCA-associated vasculitis, rituximab in combination with high-dose glucocorticoid regimens has been shown in randomized clinical trials to be noninferior to cyclophosphamide when used for remission induction therapy^11,12^ and superior to azathioprine when used for remission maintenance therapy.^13,14^ Serious infections and deaths among patients receiving rituximab therapy were similar to those observed with cyclophosphamide induction therapy and azathioprine maintenance therapy. In a single-center cohort study^15^ involving 114 patients with granulomatosis with polyangiitis, the serious infection rate was 4.9 per 100 patient-years with the use of rituximab as induction and maintenance therapy. However, the median age of participants was 52 years, and only 11%were 75 years or older.^15^ Older patients have also been underrepresented in most randomized clinical trials of individuals with ANCA-associated vasculitis.^13,16,17,18,19,20,21,22^ Only the CORTAGE (Treatment of Necrotizing Vasculitides for Patients Older Than 65 Years) clinical trial^23^ was specifically designed for the older population, although comorbidities among patients in that study were low, and no data on functional ability were assessed. Generalization of these results is therefore difficult in this population.^24^ We aimed to describe outcomes and adverse events associated with the use of rituximab as remission induction therapy and/or maintenance therapy among patients 75 years and older with ANCA-associated vasculitis.

## Methods

The ethics review committee of Hôpital Cochin approved this study. All participants provided verbal informed consent, and patients included in the French Vasculitis Study Group registry provided written informed consent. The study adhered to the Declaration of Helsinki^25^ and followed the Strengthening the Reporting of Observational Studies in Epidemiology (STROBE) reporting guideline for cohort studies.^26^

### Study Design

This multicenter cohort study used data from a prospectively collected database enriched with information from a call for observation with a retrospective data collection.^7^ Data from patients 65 years and older with ANCA-associated vasculitis were extracted from the French Vasculitis Study Group registry, in which all consecutive patients with incident ANCA-associated vasculitis from January 1, 2000, to July 1, 2018, were included. In addition, data were obtained from a call for observation of patients 75 years and older sent to French Vasculitis Study Group members on June 6, 2019. Patients were included if they had a diagnosis of granulomatosis with polyangiitis or microscopic polyangiitis according to criteria from the 2012 revised Chapel Hill Consensus Conference,^1^ received at least 1 infusion of rituximab as induction and/or maintenance therapy after age 75 years, and were either followed up for at least 6 months or deceased. Patients were excluded if they had missing data on date of birth, ANCA status, or relevant clinical manifestations.

### Classification

Classification of ANCA-associated vasculitis was performed according to European Medicines Agency classification criteria.^27^ For diagnoses that did not fit the European Medicines Agency algorithm, 2 experts on vasculitis (L.G. and X.P.) reviewed each case based on information from the entire follow-up period.

### Definitions

Follow-up started at the first infusion of rituximab as induction and/or maintenance therapy. Remission was defined as the complete absence of active clinical disease, with a Birmingham Vasculitis Activity Score (version 3; range, 0-63 points, with higher scores indicating more disease activity) of 0 points^28,29^ and a stable daily dose of prednisone of 7.50 mg or less according to recommendations from the European Alliance of Associations for Rheumatology.^30^ Serious infections were defined as those that were fatal, required hospitalization, or required administration of intravenous antibiotic medication.

### Induction and Maintenance Therapy

The remission induction regimen consisted of 4 weekly rituximab infusions of 375 mg/m^2^ of body surface area or 2 infusions of 1000 mg administered 2 weeks apart. Other immunosuppressant therapies could be prescribed. For maintenance therapy, a rituximab infusion of 500 mg was administered 4 or 5 times, with each infusion spaced 6 months apart.^17^ The protocol for induction therapy was considered complete if all infusions (4 infusions of 375 mg/m2 or 2 infusions of 1000 mg) were administered. The protocol for maintenance therapy was considered complete if all 4 or 5 infusions were administered. Otherwise, the protocol was considered discontinued.

### Data Collection

Clinical data were collected on age, sex, comorbidities, functional status, organ involvement, date of diagnosis, and date of first rituximab infusion. Data on race and ethnicity were not reported because inclusion of this information was not authorized by the ethics committee. The patient’s ANCA status was determined using immunofluorescence-linked and/or enzyme-linked immunosorbent assays at diagnosis or after diagnosis if not initially available and yielding a positive test result. The 1996 and revised 2011 Five-Factor Score (range, 0-5 points with a score of 2 when 2 or more points were present using the 1996 Five-Factor Score, and 0-4 points with a score of 2 when 2 or more points were present using the revised 2011 Five-Factor Score) and Birmingham Vasculitis Activity Score were calculated.^28,29,31,32^ Data on remission induction therapy, maintenance therapy, and glucocorticoid doses as well as the occurrence of remission, relapse, death, and serious infections were collected. Functional ability was evaluated using the Katz Activities of Daily Living score^33^ (range, 0-6 points, with 0 indicating the need for assistance with all activities of daily living and 6 indicating no need for assistance with activities of daily living). The patient’s ability to walk alone and living situation (help at home or nursing home) were assessed.

### Outcomes

We evaluated the occurrence of remission, minor and major relapses, drug discontinuation, death, and serious infections (including types of serious infections). For the analysis of outcomes among those receiving rituximab as induction therapy, patients who survived and did not experience an incident case (ie, serious infection or relapse) were censored 6 months after the first rituximab infusion. This cutoff was chosen because it would not overlap with the outcomes of maintenance therapy and because follow-up in randomized clinical trials of rituximab as induction therapy lasted for 6 months.^11,34^ For patients receiving rituximab as maintenance therapy, data were censored 28 months after the first infusion of rituximab as maintenance therapy. This cutoff was chosen to analyze outcomes associated with rituximab therapy during its immunosuppressant activity period and to allow comparison of our results with findings from randomized clinical trials.^13,17,18^

### Statistical Analysis

Categorical variables (expressed as numbers with percentages) were compared using a Fisher exact or χ^2^ test, and continuous variables (expressed as medians with IQRs) were compared using a Mann-Whitney *U* or *t* test, as appropriate. Median total follow-up was estimated using the reverse Kaplan-Meier method and defined as the time between the first infusion of rituximab and the last visit or death, whichever occurred first. Relapse, death, and serious infection rates were calculated by dividing the first incident cases occurring during follow-up by the person-time at risk and were reported as the number of events per 100 person-years (with 95% CIs). Survival curves and time to first serious infection were presented using Kaplan-Meier curves and cumulative incidence functions.^35,36^ Time zero was defined as the first infusion of rituximab as induction therapy or the first infusion of rituximab as maintenance therapy. Statistical analyses were performed using R software, version 4.0.2 (R Foundation for Statistical Computing). All statistical tests were 2-tailed, with *P* < .05 considered statistically significant.

## Results

### Patient Characteristics

**Figure 1.**
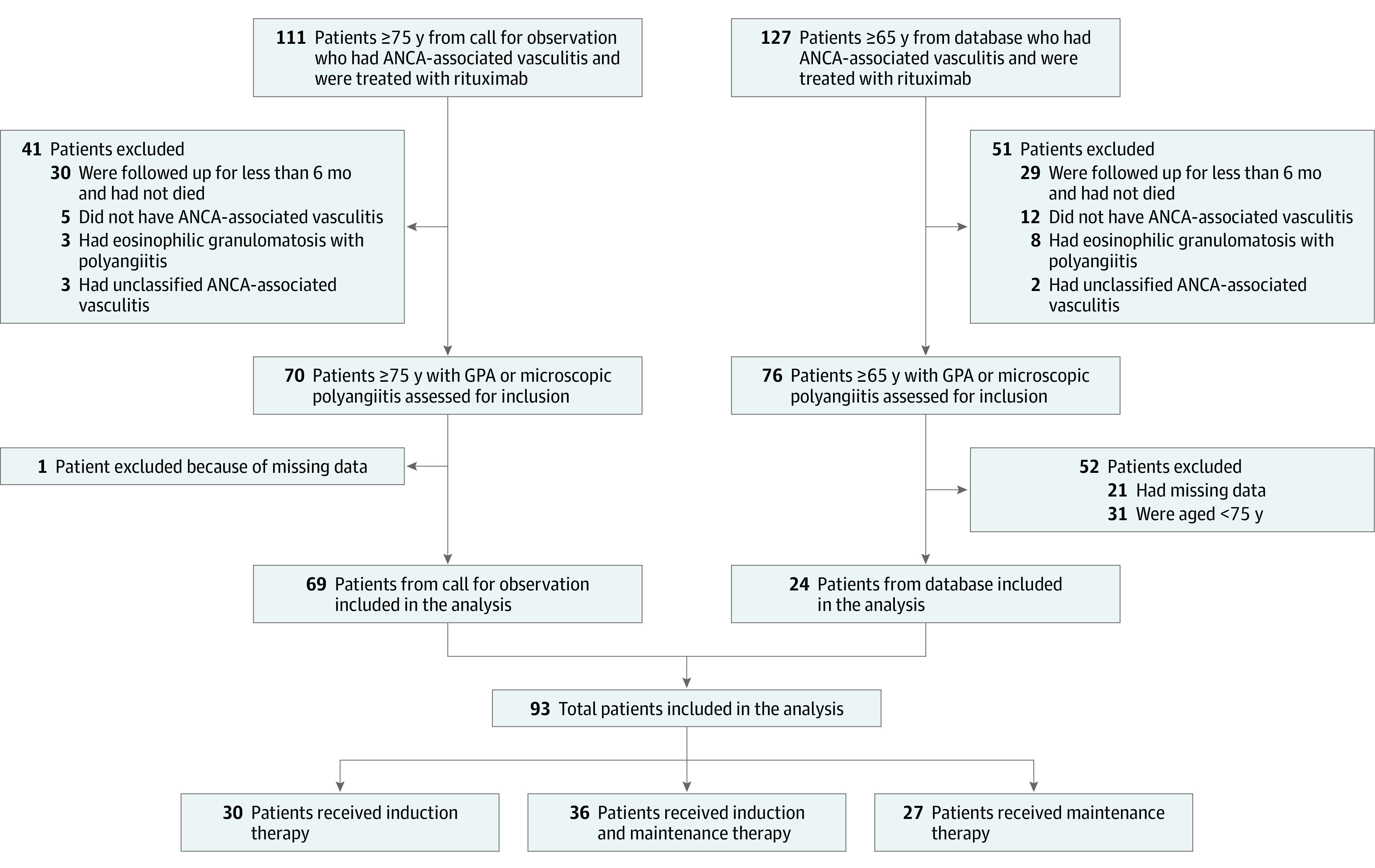
Flowchart ANCA indicates antineutrophil cytoplasmic antibody; and GPA, granulomatosis with polyangiitis.

Among 93 patients, the median (IQR) age at first rituximab infusion was 79.4 (76.7-83.1) years; 51 patients (54.8%) were women, and 42 (45.2%) were men (Figure 1). A total of 52 patients (55.9%) had a diagnosis of granulomatosis with polyangiitis, and 41 patients (44.1%) had a diagnosis of microscopic polyangiitis. Baseline characteristics at the first rituximab infusion are shown in Table 1. Overall, 30 patients (32.3%) received rituximab as induction therapy, 27 (29.0%) received rituximab as maintenance therapy, and 36 (38.7%) received rituximab as both induction and maintenance therapy. The median (IQR) total follow-up was 2.3 (1.1-4.0) years.

**Table 1.** Characteristics of Patients 75 Years and Older With Microscopic Polyangiitis or Granulomatosis With Polyangiitis Who Received Rituximab Therapy

Characteristic	Patients, No./total No. (%) (N = 93)
Age, median (IQR), y	79.4 (76.7-83.1)
Sex	
Male	42/93 (45.2)
Female	51/93 (54.8)
Weight, median (IQR), kg^a^	67 (56-74)
GPA	52/93 (55.9)
MPA	41/93 (44.1)
New-onset ANCA-associated vasculitis	82/93 (88.2)
Relapsing ANCA-associated vasculitis	11/93 (11.8)
ANCA positivity (IF and/or ELISA)	91/93 (97.8)
PR3-ANCA	36/93 (38.7)
MPO-ANCA	51/93 (54.8)
PR3-ANCA plus MPO-ANCA	3/93 (3.2)
Comorbidities	
Hypertension	60/92 (65.2)
Ischemic heart disease	8/92 (8.7)
Atrial fibrillation	14/93 (15.1)
Diabetes	18/92 (19.6)
Cancer (active or past)	15/91 (16.5)
Osteoporosis	10/93 (10.8)
Chronic bronchitis	4/93 (4.3)
Ischemic stroke	7/93 (7.5)
Chronic kidney disease	4/93 (4.3)
Functional ability	
ADL score, median (IQR)^b^	6 (6-6)
Walks alone	44/53 (83.0)
Requires help at home	4/53 (7.5)
Lives in nursing home	1/63 (1.6)
Clinical manifestations	
Fever	22/90 (24.4)
Weight loss	38/89 (42.7)
Myalgia	16/89 (18.0)
Arthralgia or arthritis	22/91 (24.2)
Cutaneous	12/92 (13.0)
Ophthalmologic	7/92 (7.6)
Ear, nose, and throat	35/92 (38.0)
Pulmonary	44/92 (47.8)
Cardiomyopathy	3/91 (3.3)
Gastrointestinal	2/92 (2.2)
Kidney involvement	70/92 (76.1)
Peripheral nervous system	17/92 (18.5)
Central nervous system	4/92 (4.3)
Biological findings	
Creatinine level, median (IQR), mg/dL^c^	2.04 (1.02-3.44)
Creatinine level ≥1.58 mg/dL	53/90 (58.9)
C-reactive protein, median (IQR), mg/dL^d^	7.70 (2.36-11.35)
Lymphocytes, median (IQR), cells/mm^3^^e^	1280 (907-1504)
γ-Globulin concentration, median (IQR), g/dL^f^	1.02 (0.63-1.26)
BVAS, median (IQR)^g^	14 (10-19)
FFS (1996)^h^	
0	31/91 (34.1)
1	33/91 (36.3)
2	27/91 (29.7)
FFS (2011)	
1	27/91 (29.7)
2	64/91 (70.3)

Abbreviations: ADL, Activities of Daily Living scale; ANCA, antineutrophil cytoplasmic antibody; BVAS, Birmingham Vasculitis Activity Score; ELISA, enzyme-linked immunosorbent assay; FFS, Five-Factor Score; GPA, granulomatosis with polyangiitis; IF, indirect immunofluorescence; MPA, microscopic polyangiitis; MPO, myeloperoxidase; PR3, proteinase 3.

SI conversion factors: To convert C-reactive protein from milligrams per deciliter to milligrams per liter, multiply by 10; to convert creatinine from milligrams per deciliter to micromoles per liter, multiply by 88.4; to convert γ-globulin from grams per deciliter to grams per liter, multiply by 10.

^a^ Data were missing for 25 patients.

^b^ Score range, 0-6 points, with 0 indicating the need for assistance with all activities of daily living and 6 indicating no need for assistance with activities of daily living. Data were missing for 45 patients.

^c^ Data were missing for 8 patients.

^d^ Data were missing for 49 patients.

^e^ Data were missing for 63 patients.

^f^ Data were missing for 69 patients.

^g^ Score range for version 3, 0-63 points, with higher scores indicating more disease activity. Data were missing for 2 patients.

^h^ Score range, 0-5 points with a score of 2 when 2 or more points were present using the 1996 Five-Factor Score, and 0-4 points with a score of 2 when 2 or more points were present using the revised 2011 Five-Factor Score. Data were missing for 2 patients.

### Rituximab as Remission Induction Therapy

Characteristics of the 66 patients who received rituximab as induction therapy (which included those who received it as induction therapy only or before receiving it as maintenance therapy) are shown in eTable 1 in Supplement 1. The median (IQR) age at the first rituximab induction infusion was 79.7 (76.5-83.2) years. Remission was achieved in 57 patients (86.4%), and 2 patients (3.0%) experienced relapse. A total of 58 patients (87.9%) completed the treatment protocol. Reasons for early discontinuation of the protocol included death before the last rituximab infusion (6 patients [9.1%]) and being considered too fragile based on practitioner assessment (2 patients [3.0%]). All patients received high-dose glucocorticoid regimens in combination with rituximab, with an initial daily dose of 1.00 mg/kg. In addition to rituximab and glucocorticoid regimens, 18 patients (27.3%) received at least 1 additional therapy; 9 patients received cyclophosphamide, 10 received plasma exchange, 2 received methotrexate, and 1 received an infusion of immunoglobulins.

**Figure 2.**
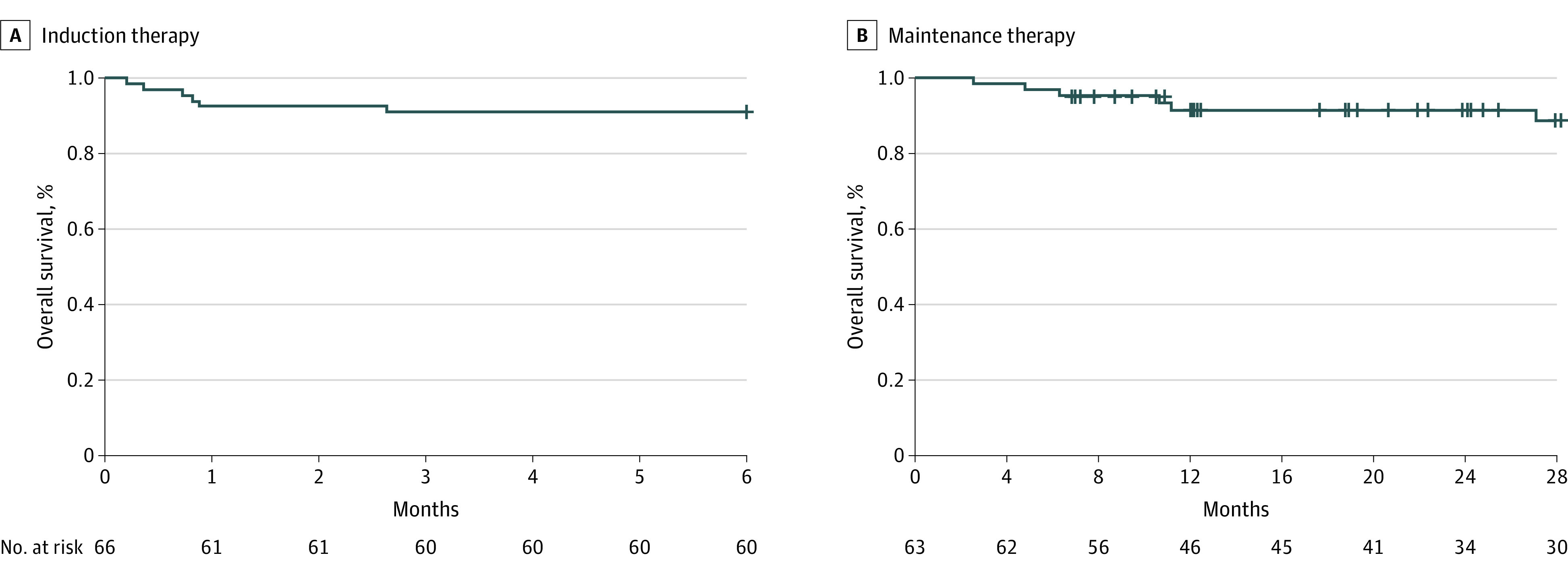
Overall Survival Among Patients 75 Years and Older With ANCA-Associated Vasculitis Who Received Rituximab Therapy Kaplan-Meier estimation. ANCA indicates antineutrophil cytoplasmic antibody.

Death occurred in 6 patients (9.1%), with an incidence of 19.7 (95% CI, 7.2-42.9) per 100 patient-years (Figure 2). Among the 25 patients with available data, the median (IQR) CD19^+^ lymphocyte count at 6 months after the first rituximab infusion was 0 (0-9) per mm^3^, and the median (IQR) γ-globulin concentration was 0.59 (0.46-0.78) g/dL (to convert grams per deciliter to grams per liter, multiply by 10).

### Rituximab as Maintenance Therapy

Baseline characteristics of the 63 patients who received rituximab as maintenance therapy (which included those who received it as maintenance therapy only or in addition to receiving it as induction therapy) are shown in eTable 1 in Supplement 1. The median (IQR) age was 79.4 (77.2-83.3) years at the first maintenance rituximab infusion. Previous remission induction treatments and glucocorticoid tapering are shown in Table 2. A total of 36 patients (57.1%) completed the protocol. Reasons for early discontinuation before the last rituximab infusion included unavailability for follow-up before the last infusion (11 patients [17.5%]), immunosuppression sparing (7 patients [11.1%]), inclusion in the MAINRITSAN 2 (Comparison Study of Two Rituximab Regimens in the Remission of ANCA Associated Vasculitis) clinical trial (5 patients [7.9%]),^17^ occurrence of relapse (1 patient [1.6%]), and death before the last infusion (3 patients [4.8%]).

**Table 2.** Induction Therapy and Glucocorticoid Tapering Administered Before Rituximab for Maintenance Therapy

Treatment	Patients, No. (%) (n = 63)
Induction therapy before receipt of rituximab as maintenance therapy	
Cyclophosphamide	33 (52.4)
Rituximab	36 (57.1)
Plasma exchange	12 (19.0)
Methotrexate	2 (3.2)
Glucocorticoids alone	0
Glucocorticoid tapering, median (IQR), d	
Time to reach 20 mg/d^a^	102.0 (83.0-119.7)
Time to reach 10 mg/d^b^	163.0 (132.2-191.5)
Glucocorticoid duration^c^	629.0 (297.8-877.8)

^a^ Data were missing for 11 patients.

^b^ Data were missing for 13 patients.

^c^ Data were missing for 7 patients.

Death within 28 months occurred in 6 patients (9.5%), with an incidence rate of 5.3 (95% CI, 1.9-11.6) per 100 patient-years (Figure 2). Relapse occurred in 2 patients (3.2%), with an incidence rate of 1.8 (95% CI, 0.2-6.5) per 100 patient-years. Among the 27 patients with available data, the median (IQR) CD19^+^ lymphocyte count at 6 months after the last rituximab infusion was 0 (0-1) per mm^3^, and the median (IQR) γ-globulin concentration was 0.52 (0.50-0.64) g/dL.

### Serious Infections During Receipt of Rituximab Therapy

**Figure 3.**
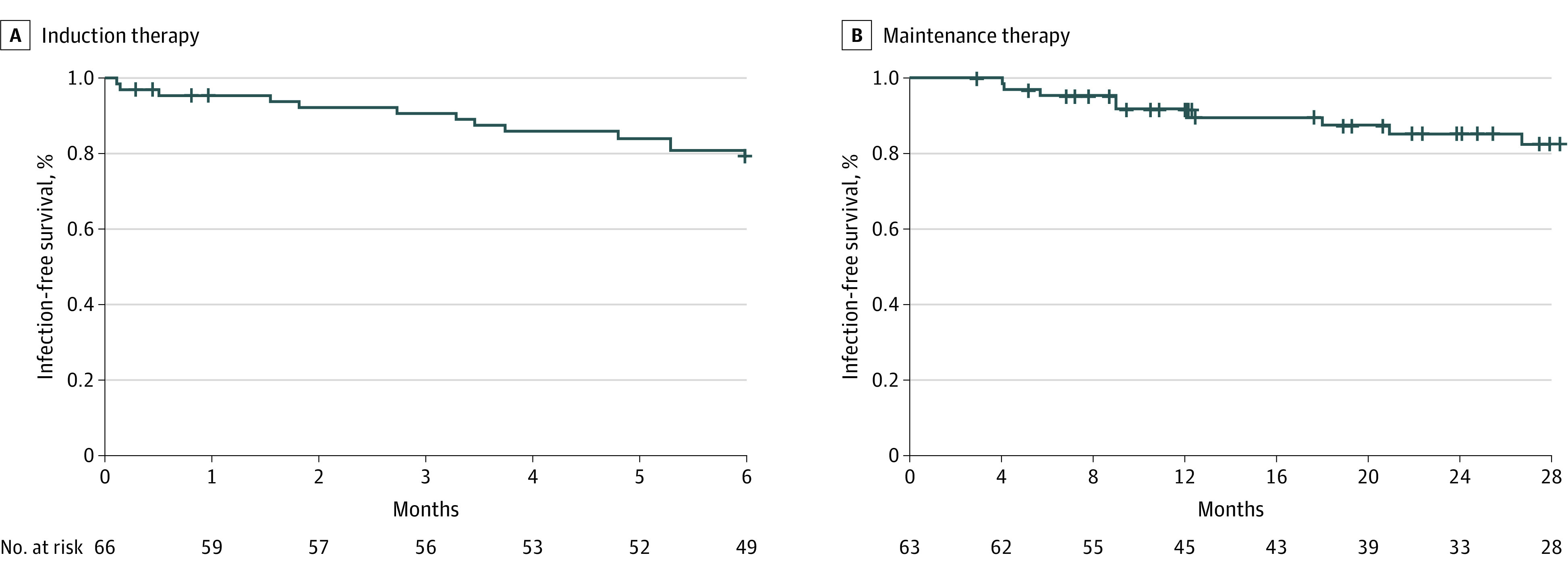
Infection-free Survival Among Patients 75 Years and Older With ANCA-Associated Vasculitis Who Received Rituximab Therapy ANCA indicates antineutrophil cytoplasmic antibody.

The incidence of serious infections was significantly higher among patients who received rituximab as induction therapy vs maintenance therapy. Serious infections within 6 months after the first infusion of rituximab as induction therapy were observed in 13 of 66 patients (19.7%), with an incidence rate of 46.6 (95% CI, 24.8-79.7) per 100 patient-years. Among patients who received rituximab as maintenance therapy, serious infections occurred in 9 of 63 patients (14.3%) within 28 months, with an incidence rate of 8.4 (95% CI, 3.8-15.9) per 100 patient-years (*P* = .004 for comparison with induction therapy). Infection-free survival rates among patients who received rituximab as both induction and maintenance therapy are shown in Figure 3, and cumulative incidence rates of serious infections and death are shown in the eFigure in Supplement 1. The 6-month infection-free survival rate was 0.79 (95% CI, 0.70-0.90) among patients receiving rituximab as induction therapy and 0.95 (95% CI, 0.90-1.00) among those receiving rituximab as maintenance therapy. Serious infections that occurred among patients who received rituximab as both induction and maintenance therapy are shown in eTable 2 and eTable 3 in Supplement 1. Most infections (12 of 22 [54.5%]) were gram-negative bacterial infections reflecting neutrophil- and monocyte- or macrophage-related immunodeficiency.^37^

Among patients receiving rituximab as induction therapy, the incidence rate of serious infections in those who initially received 1.00 mg/kg or more of glucocorticoids daily was 55.1 (95% CI, 27.5-98.6) per 100 patient-years. Patients who initially received doses lower than 1.00 mg/kg (median [IQR], 0.60 [0.33-0.75] mg/kg) had an incidence rate of 33.7 (95% CI, 4.1-121.7) serious infections per 100 patient-years, with no significant difference compared with those who received doses of 1.00 mg/kg or more (*P* = .46). The baseline γ-globulin concentrations and lymphocyte levels did not differ between patients with vs without a serious infection (γ-globulin concentration: median [IQR], 0.82 [0.60-1.05] g/dL vs 1.02 [0.66-1.26] g/dL; *P* = .59; lymphocytes: median [IQR], 1440 [880-1688] cells per mm^3^ vs 1270 [955-1450] cells per mm^3^; *P* = .75).

## Discussion

This large cohort study of patients 75 years and older who received rituximab for the treatment of ANCA-associated vasculitis found that rituximab therapy was associated with achievement and maintenance of remission in most patients. The occurrence of serious infections was high during induction therapy. Most infections reflected neutrophile- and monocyte- or macrophage-related immunodeficiency and occurred during the first months of induction therapy, which could suggest involvement of the concomitant high dose of glucocorticoids.

Few studies have evaluated the efficacy and tolerability of rituximab therapy in the older population. A prospective cohort study^38^ involving 191 patients with rheumatoid arthritis who received rituximab therapy reported serious infections in 26.5% of those 75 years and older, which occurred more frequently than in younger patients. A retrospective study^39^ of 31 patients 60 years and older with ANCA-associated vasculitis who received rituximab therapy observed that rituximab could induce remission, but 16% of patients had serious infections within the first year. To our knowledge, the current study was the first to assess outcomes and adverse events associated with the use of rituximab therapy among a large cohort of older patients with ANCA-associated vasculitis.

Our study found a high incidence rate of serious infections (46.6 per 100 patient-years) among patients who received rituximab as induction therapy. However, the incidence rate of serious infections (8.4 per 100 patient-years) among those who received rituximab as maintenance therapy did not significantly increase compared with the incidence rate reported in 2 prospective cohort studies from France^15^ and the US^40^ involving younger patients with ANCA-associated vasculitis who received treatment with rituximab. The French study^15^ reported an incidence rate of 4.9 infections per 100 patient-years, and the US study^40^ reported 7.1 infections per 100 patient-years. A US cohort study^41^ involving 188 patients noted that one-third of infections occurred within 4 weeks of the first induction dose of rituximab. A prospective European cohort study^42^ of patients with ANCA-associated vasculitis found that infections, more than disease activity, were associated with an increased risk of mortality during the first year of therapy. Thus, efforts might focus on preventing the occurrence of serious infections during the first months of rituximab induction therapy, particularly in older populations with limited functional ability.

It is likely that the observed high incidence of infections during rituximab induction therapy was associated with its combination with high-dose glucocorticoid regimens. We found that most serious infections were gram-negative bacterial infections that reflected neutrophile- and monocyte- or macrophage-related immunosuppression.^37^ These results were similar to previously published findings.^15,41,43^ Our data suggest a need for better prophylactic measures and perhaps more parsimonious use of glucocorticoids in this older population. One study^44^ found that an intravenous pulse of methylprednisolone also increased the risk of serious infections among patients aged 62 years with ANCA-associated vasculitis. The results of the CORTAGE randomized clinical trial^23^ showed that using an induction regimen based on a lower cumulative dose of glucocorticoids and cyclophosphamide in patients 65 years and older could decrease the occurrence of serious adverse events, including serious infections. The PEXIVAS (Plasma Exchange and Glucocorticoid Dosing in the Treatment of Anti-neutrophil Cytoplasm Antibody Associated Vasculitis) randomized clinical trial^21^ also demonstrated that a glucocorticoid regimen with a reduced dose decreased the 1-year risk of serious infections and was noninferior to a standard-dose regimen with respect to the risk of death or end-stage kidney disease. Another open-label randomized clinical trial^34^ has shown that, among patients with nonsevere ANCA-associated vasculitis who received rituximab therapy, a reduced-dose glucocorticoid regimen was noninferior to a conventional high-dose glucocorticoid regimen with respect to the disease remission rate at 6 months but led to significantly fewer serious adverse events and serious infections. This decreased glucocorticoid regimen likely needs to be evaluated specifically in older patients with ANCA-associated vasculitis. Future studies are warranted to find the best balance between limiting the occurrence of serious infections and maintaining sufficient benefit with regard to ANCA-associated vasculitis activity.

Age itself is a risk factor for serious infections among patients with ANCA-associated vasculitis, even when those patients receive treatment with drugs other than rituximab.^5,6,45^ In a cohort study^9^ of 83 patients with ANCA-associated vasculitis who had a median age of 74 years and were followed up for 3 years, 44 infections occurred and were the most common type of adverse event, even though rituximab therapy was administered to only 37% of patients. In a study^10^ evaluating long-term outcomes of patients with a median age of 72 years and ANCA-associated vasculitis with kidney involvement, 42% of patients experienced a serious infection within the first 6 months after diagnosis, one-half of which occurred during the first month. In this population, only 7% of patients received rituximab as induction therapy.^10^

Few studies have evaluated the association of parameters specific to older patients, such as functional ability, frailty, and comorbidities, with outcomes. Worse performance on the Rockwood Clinical Frailty Scale was associated with lower overall survival in a cohort of 83 patients with ANCA-associated vasculitis and a median age of 74 years.^9^ Patients with low handgrip strength (an important marker of nutritional status and a component of the Fried Frailty Phenotype^46^) had a significantly higher score on the Vasculitis Damage Index and an increased risk of serious adverse events.^47^ More studies are needed to better define patients who are most at risk of experiencing an adverse outcome. Future studies involving this older population could incorporate evaluations of the following areas: functional status (eg, using the Activities of Daily Living scale^33^ or the Instrumental Activities of Daily Living scale^48^), nutritional status (eg, using body mass index or percentage of weight loss), cognitive function (eg, using the Montreal Cognitive Assessment^49^), frailty (eg, using the Fried Frailty Phenotype^46^ or the Rockwood Clinical Frailty Scale^50^), and gait (eg, using the Short Physical Performance Battery^51^).

### Strengths and Limitations

This study has several strengths. To our knowledge, it is the first large cohort study of patients with ANCA-associated vasculitis who received rituximab therapy after age 75 years in a real-life setting. We provide data on outcomes associated with rituximab among an older population in whom no specific clinical trials are likely to be performed. Comorbidities, functional ability, clinical and biological characteristics, and outcomes were precisely described for both induction and maintenance therapies. Analysis was performed at 6 months and 28 months after the first infusion, but median follow-up was more than 2 years.

The study also has limitations. We did not perform a comparison with a control group, such as patients who received cyclophosphamide and azathioprine. However, the inclusion of a control group of patients receiving azathioprine as maintenance therapy could raise concern because randomized clinical trials^13,14^ have shown that rituximab is superior to azathioprine in preventing relapse with no increase in toxic effects, and rituximab is now the criterion standard for maintenance therapy.

## Conclusions

In this cohort study, most patients 75 years and older who received rituximab therapy achieved sustained remission and did not experience relapse. However, a high incidence rate of serious infections was observed when rituximab was used to induce remission in combination with high-dose glucocorticoid regimens. Future efforts might focus on finding the best induction regimen to reduce infections without limiting benefits in this population, including evaluation of decreased glucocorticoid regimens specifically among older patients with ANCA-associated vasculitis.

## Notes

Supplement 1. **eTable 1.** Characteristics of Patients 75 Years and Older With GPA or MPA Treated With Rituximab for Induction or Maintenance Therapy **eTable 2.** Description of the 13 Serious Infections Among Patients Treated With Rituximab as Induction Therapy **eTable 3.** Description of the 9 Serious Infections Among Patients Treated With Rituximab as Maintenance Therapy **eFigure.** Cumulative Incidence of Infection and Death Among Patients 75 Years and Older With ANCA-Associated Vasculitis Treated With Rituximab as Induction Therapy or Maintenance Therapy (Cumulative Incidence Function)

Supplement 2. **Nonauthor Collaborators.** The French Vasculitis Study Group
