# Supplementary material for: Evaluation of Rituximab for Induction and Maintenance Therapy in Patients 75 Years and Older With Antineutrophil Cytoplasmic Antibody–Associated Vasculitis
Source: JAMA Netw Open. 2022 Jul 8;5(7):e2220925. doi: 10.1001/jamanetworkopen.2022.20925 (PMC9270693; doi:10.1001/jamanetworkopen.2022.20925)
Supplement: Supplement 1. — eTable 1. Characteristics of Patients 75 Years and Older With GPA or MPA Treated With Rituximab for Induction or Maintenance Therapy eTable 2. Description of the 13 Serious Infections Among Patients Treated With Rituximab as Induction Therapy eTable 3. Description of the 9 Serious Infections Among Patients Treated With Rituximab as Maintenance Therapy eFigure. Cumulative Incidence of Infection and Death Among Patients 75 Years and Older With ANCA-Associated Vasculitis Treated With Rituximab as Induction Therapy or Maintenance Therapy (Cumulative Incidence Function) [file jamanetwopen-e2220925-s001.pdf]

Supplementary Online Content

Thietart S, Karras A, Augusto JF, et al; French Vasculitis Study Group. Evaluation of rituximab for induction and maintenance therapy in patients 75 years and older with antineutrophil cytoplasmic antibody–associated vasculitis. *JAMA Netw Open*. 2022;5(7):e2220925. doi:10.1001/jamanetworkopen.2022.20925

eTable 1. Characteristics of Patients 75 Years and Older With GPA or MPA Treated With Rituximab for Induction or Maintenance Therapy

eTable 2. Description of the 13 Serious Infections Among Patients Treated With Rituximab as Induction Therapy

eTable 3. Description of the 9 Serious Infections Among Patients Treated With Rituximab as Maintenance Therapy

eFigure. Cumulative Incidence of Infection and Death Among Patients 75 Years and Older With ANCA-Associated Vasculitis Treated With Rituximab as Induction Therapy or Maintenance Therapy (Cumulative Incidence Function)

This supplementary material has been provided by the authors to give readers additional information about their work.

© 2022 Thietart S et al. *JAMA Network Open*.

eTable 1. Characteristics of Patients 75 Years and Older With GPA or MPA Treated With Rituximab for Induction or Maintenance Therapy

	**Induction therapy**	**Maintenance therapy**	**Missing values**
**Number**	66	63	
Age	79.7 [76.5-83.2]	79.4 [77.2-83.3]	0
Male	28 (42)	29 (46)	0
Weight (kg)	65 [55-71]	67 [56-78]	35
GPA	41 (62)	33 (52)	0
MPA	25 (38)	30 (48)	0
New-onset AAV	55 (83)	56 (89)	0
Relapsing AAV	11 (17)	7 (11)	0
Positive ANCA (IF and/or ELISA)	65 (98)	61 (97)	0
Anti-PR3 ANCA	28 (42)	22 (35)	0
Anti-MPO ANCA	34 (52)	37 (59)	0
Anti-PR3 + anti-MPO ANCA	2 (3)	2 (3)	0
ANCA- (ELISA)	2 (3)	2 (3)	0
**Comorbidities**			
Hypertension	40 (62)	45 (71)	1
Ischemic heart disease	3 (5)	6 (10)	1
Atrial fibrillation	10 (15)	9 (14)	0
Diabetes	12 (18)	13 (21)	1
Cancer (active or past)	11 (17)	11 (18)	2
Osteoporosis	8 (12)	5 (8)	0
Chronic bronchitis	3 (5)	3 (5)	0
Ischemic stroke	3 (5)	5 (8)	0
Chronic kidney disease	3 (5)	3 (5)	0
**Functional ability**			
ADL	6 [6-6]	6 [6-6]	62
Walks alone	30 (83)	31 (89)	58
Requires help at home	3 (8)	3 (8)	54
Lives in nursing home	1 (2)	0	39
**Clinical manifestations**			
Fever	14 (22)	12 (19)	3
Weight loss	23 (37)	26 (42)	4
Myalgia	8 (13)	10 (16)	4
Arthralgia/arthritis	12 (19)	15 (24)	3
Cutaneous	6 (9)	8 (13)	1
Ophthalmologic	6 (9)	5 (8)	1
Ear, nose, throat	27 (42)	24 (38)	1
Pulmonary	30 (46)	30 (48)	1
Cardiomyopathy	2 (3)	1 (2)	2
Gastrointestinal	1 (2)	1 (2)	1
Renal involvement	50 (77)	45 (71)	1
Peripheral nervous system	13 (20)	11 (17)	2
Central nervous system	2 (3)	4 (6)	1

© 2022 Thietart S et al. *JAMA Network Open*.

**Biological findings**			
Creatinine level (µmol/L)	183 [101-317]	197 [105-307]	13
Creatinine level ≥140 µmol/L	36 (57)	35 (56)	4
CRP at diagnosis (mg/L)	80 [23-117]	87 [30-141]	43
Lymphocyte (/mL)	1,290 [910-1,576]	1,270 [1,000-1,630]	84
Gammaglobulin (g/L)	10.6 [6.6-12.9]	5.7 [4.6-7.2]	84
**Scores**			
BVAS at diagnosis	14 [9.5-18]	14 [9-18]	1
FFS 1996			2
0	24 (37.5)	23 (36)	
1	24 (37.5)	22 (35)	
2	16 (25)	18 (29)	
FFS 2011			2
1	20 (31)	21 (33)	
2	44 (69)	42 (67)	

Continuous variables are expressed as median [1st–3rd quartiles], and categorical variables as sample size (percentages). AAV: ANCA-associated vasculitis; ADL: Katz's Activity of Daily Living; ANCA: antineutrophil cytoplasmic antibodies; BVAS: Birmingham Vasculitis Activity Score; CRP: C reactive protein; ELISA: enzyme-linked immunosorbent assay; FFS: Five Factor Score; GPA: granulomatosis with polyangiitis; IF: indirect immunofluorescence; MPA: microscopic polyangiitis; MPO: myeloperoxidase; PR3: proteinase 3.

© 2022 Thietart S et al. *JAMA Network Open*.

eTable 2. Description of the 13 Serious Infections Among Patients Treated With Rituximab as Induction Therapy

Immunological deficiency	Pathogen Type of infection	Initial glucocorticoids dose (mg/kg/day)	Concomitant therapy
Neutrophil- & monocyte/macrophage-mediated immunodeficiency	Gram-negative pyelonephritis	0.6	0
	Nocardia pneumonia	1.4	0
	Gram-negative pyelonephritis	1	Plasma exchange
	Gram-negative bacteriemia	1	0
	Gram-negative pyelonephritis	1	0
	Gram-negative pneumonia	1	Plasma exchange
	Gram-positive and gram-negative osteitis	1	0
	Pseudomonas aeruginosa pneumonia & bacteriemia	1	0
	Pseudomonas aeruginosa septic arthritis	1	Cyclophosphamide & plasma exchange
	Respiratory viral infection	1	0
B-lymphocyte- & humoral- mediated immunodeficiency	Encapsulated bacteria pneumonia	1	0
	Encapsulated bacteria pneumonia & septic choc	0.5	0
T-lymphocytes mediated deficiency	Ophthalmic zona	1	Methotrexate

Thirteen infections were seen in 13 patients during the rituximab induction therapy; each line represents one infection.
Treatment-related immunological deficiency are reported according to those described by Azoulay, et *al*[34]

© 2022 Thietart S et al. *JAMA Network Open*.

eTable 3. Description of the 9 Serious Infections Among Patients Treated With Rituximab as Maintenance Therapy

Immunological deficiency	Pathogen type of infection	Time to achieve 20 mg daily of prednisone-equivalent (days)	Time to achieve 10 mg daily of prednisone-equivalent (days)	Time to achieve prednisone withdrawal (days)	Previous induction therapy[a]
Neutrophil- & monocyte/macrophage-mediated immunodeficiency	Gram-negative pyelonephritis	10	MD	508	Rituximab
	Gram-negative pyelonephritis	MD	205	643	Rituximab and methotrexate
	Gram-negative cholecystitis	MD	122	1,248	Rituximab
	Gram-positive septic arthritis	50	80	394	Rituximab and plasma exchange
	Gram-negative acute cholangitis	103	MD	201	Rituximab
	Gram-positive pyelonephritis	97	157	247	Rituximab
T-lymphocytes or monocyte/macrophage mediated immunodeficiency	Influenza virus flue	93	157	251	Cyclophosphamide
B-lymphocyte- and humoral- mediated immunodeficiency	Encapsulated bacteria pneumonia	MD	652	847	Cyclophosphamide
	Encapsulated bacteria pneumonia	118	MD	MD	Cyclophosphamide

Nine serious infections were seen in 9 patients during the maintenance rituximab therapy; each line represents one infection.
[a]Treatment-related immunological deficiency are reported according to those described by Azoulay, et *al*[34]
MD: missing data.

© 2022 Thietart S et al. *JAMA Network Open*.

eFigure. Cumulative Incidence of Infection and Death Among Patients 75 Years and Older With ANCA-Associated Vasculitis Treated With Rituximab as (A) Induction Therapy or (B) Maintenance Therapy (Cumulative Incidence Function)

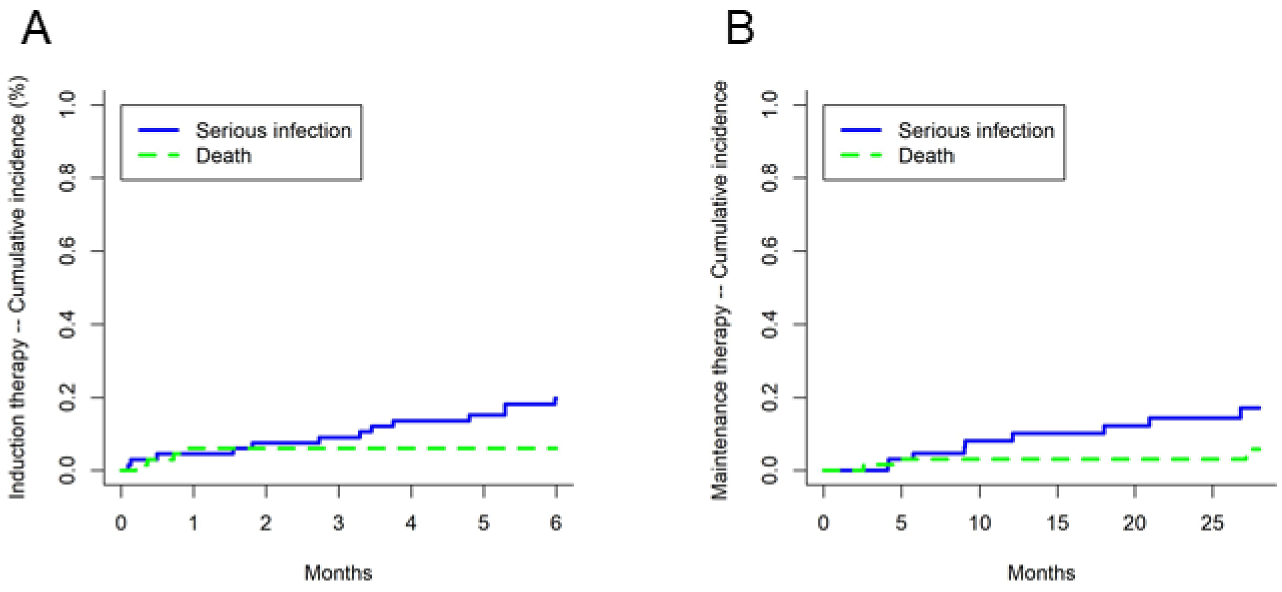

© 2022 Thietart S et al. *JAMA Network Open*.
