# Supplementary material for: Evaluation of Rituximab for Induction and Maintenance Therapy in Patients 75 Years and Older With Antineutrophil Cytoplasmic Antibody–Associated Vasculitis
Source: JAMA Netw Open. 2022 Jul 8;5(7):e2220925. doi: 10.1001/jamanetworkopen.2022.20925 (PMC9270693; doi:10.1001/jamanetworkopen.2022.20925)
Supplement: Supplement 2. — Nonauthor Collaborators. The French Vasculitis Study Group [file jamanetwopen-e2220925-s002.pdf]

Supplemental Online Content: Nonauthor Collaborators
*First name, last name, and suffix (if applicable) are required and will appear in PubMed.

*Group Name(s): The French Vasculitis Study Group							
***First Name and Middle Initial(s)**	***Last Name**	***Suffix (eg, Jr, III)**	Academic Degrees	Institution	Location (city, state/province, country)	Role or Contribution, eg, chair, principal investigator	Group (if more than 1 Group listed in the byline) and/or Subgroup (eg, Steering Committee)
Asma	Alla		MD	department of nephrology, CHU Nancy	Nancy, France	data acquisition	
Vincent	Audard		MD, PhD	department of nephrology, CHU Henri Mondor	Créteil, France	data acquisition	
Olivier	Aumaître		MD, PhD	department of internal medicine, CHU de Clermont Ferrand	Clermont Ferrand, France	data acquisition	
Stéphane	Bally		MD	department of nephrology, CH Métropole Savoie	Chambéry, France	data acquisition	
Bernard	Bonnotte		MD, PhD	department of internal medicine, CHU Dijon Bourgogne	Dijon, France	data acquisition	
Pierre	Charles		MD	department of internal medicine, Institut Mutualiste Montsouris	Paris, France	data acquisition	
Pascal	Cohen		MD	department of internal medicine, CHU Cochin	Paris, France	data acquisition	
Alice	Corbel		MD	department of nephrology, CHU Nancy	Nancy, France	data acquisition	
Maud	Cousin		MD	department of nephrology, CHU Angers	Angers, France	data acquisition	
Yoann	Cabrol		MD	department of internal medicine, CH Bretagne Atlantique	Vannes, France	data acquisition	
Stéphane	Durupt		MD	department of internal medicine, CH Lyon-Sud	Lyon, France	data acquisition	

Supplemental Online Content: Nonauthor Collaborators
*First name, last name, and suffix (if applicable) are required and will appear in PubMed.

***First Name and Middle Initial(s)**	***Last Name**	***Suffix (eg, Jr, III)**	Academic Degrees	Institution	Location (city, state/province, country)	Role or Contribution, eg, chair, principal investigator	Group (if more than 1 Group listed in the byline) and/or Subgroup (eg, Steering Committee)
Benoit	Faucher		MD	department of internal medicine, CHU Marseille	Marseille, France	data acquisition	
Ségolène	Gendreau		MD	department of internal medicine, CHU Henri Mondor	Créteil, France	data acquisition	
Nicolas	Girszyn		MD	department of internal medicine, CHU Rouen	Rouen, France	data acquisition	
Pascal	Godmer		MD	department of internal medicine, CH Bretagne Atlantique	Vannes, France	data acquisition	
Mohamed	Hamidou		MD PhD	department of internal medicine, CHU Nantes	Nantes, France	data acquisition	
Noémie	Jourde-Chiche		MD PhD	department of nephrology, CHU Marseille La Conception	Marseille, France	data acquisition	
Adeline	Lacraz		MD	department of nephrology, CH de la Côte Basque	Bayonne, France	data acquisition	
Cédric	Landron		MD	department of internal medicine, CH du Haut Anjou	Château-Gontier-sur-Mayenne, France	data acquisition	
Claire	Le Jeunne		MD	department of internal medicine, CHU Cochin	Paris, France	data acquisition	
François	Lifermann		MD	department of internal medicine, CH de Dax	Dax, France	data acquisition	
Eric	Liozon		MD	department of internal medicine, CHU de Limoges	Limoges, France	data acquisition	

Supplemental Online Content: Nonauthor Collaborators
*First name, last name, and suffix (if applicable) are required and will appear in PubMed.

***First Name and Middle Initial(s)**	***Last Name**	***Suffix (eg, Jr, III)**	Academic Degrees	Institution	Location (city, state/province, country)	Role or Contribution, eg, chair, principal investigator	Group (if more than 1 Group listed in the byline) and/or Subgroup (eg, Steering Committee)
Sylvain	Marchand-Adam		MD PhD	department of pulmonology, CH Bretonneau - Tours	Tours, France	data acquisition	
François	Maurier		MD	department of internal medicine, Hôpitaux Privés de Metz	Metz, France	data acquisition	
Arsène	Mékinian		MD PhD	department of internal medicine, CHU Saint Antoine	Paris, France	data acquisition	
Christian	Pagnoux		MD, MSc, MPH	Vasculitis clinic, Mount Sinai Hospital	Toronto, Canada	data acquisition	
Thomas	Quéméneur		MD	department of internal medicine, CH Valenciennes	Valenciennes, France	data acquisition	
Viviane	Queyrel		MD	department of internal medicine, CHU de Nice	Nice, France	data acquisition	
Hanta Nirina	Rakotoarivelo		MD	department of internal medicine, Hôpital Louis Pasteur	Colmar, France	data acquisition	
Alexis	Régent		MD PhD	department of internal medicine, CHU Cochin	Paris, France	data acquisition	
Quitterie	Reynaud		MD	department of internal medicine, CH Lyon Sud	Lyon, France	data acquisition	
Virginie	Rieu		MD	department of internal medicine, CHU Clermont Ferrand	Clermont Ferrand, France	data acquisition	
Karim	Sacre		MD PhD	department of internal medicine, CHU Bichat	Paris, France	data acquisition	

Supplemental Online Content: Nonauthor Collaborators
*First name, last name, and suffix (if applicable) are required and will appear in PubMed.

***First Name and Middle Initial(s)**	***Last Name**	***Suffix (eg, Jr, III)**	Academic Degrees	Institution	Location (city, state/province, country)	Role or Contribution, eg, chair, principal investigator	Group (if more than 1 Group listed in the byline) and/or Subgroup (eg, Steering Committee)
André	Soto		MD	department of internal medicine, CH de Bigorre	Tarbes, France	data acquisition	
Dimitri	Titeca-Beauport		MD	department of nephrology, CHU Amiens	Amiens, France	data acquisition	
Stéphane	Vinzio		MD	department of internal medicine, Groupe Hospitalier Mutualiste	Grenoble, France	data acquisition	
